# Commentary: Causal associations between schizophrenia and cancers risk: a Mendelian randomization study

**DOI:** 10.3389/fonc.2024.1374235

**Published:** 2024-03-19

**Authors:** Zhe Wang, Da Li, Cheng Zhang

**Affiliations:** Department of General Surgery, General Hospital of Northern Theater Command (Formerly Called General Hospital of Shenyang Military Area), Shenyang, China

**Keywords:** schizophrenia, Mendelian randomization, cancer, causal relationship, TSMR

## Introduction

Schizophrenia is a multifaceted and incapacitating psychiatric disorder widely recognized as one of the most severe and disabling conditions globally, prompting ongoing debate and scrutiny within the medical field regarding the potential association between mental illness and tumors. In recent years, advancements in statistical methods and high-throughput sequencing technologies have enhanced the efficacy of Mendelian randomization (MR) in elucidating potential causal relationships between exposures and outcomes. In this context, we read with interest the study ‘‘Causal associations between schizophrenia and cancers risk: a Mendelian randomization study’’ by Zhou et al. (1). To our knowledge, the article by Zhou et al. (1) is the most comprehensive current MR article assessing the causal relationship between schizophrenia and cancer risk. However, the trial appeared to suffer from some data issues as well as methodological biases.

## Discussion and conclusion

First, the researchers employed genome-wide association studies (GWAS) pertaining to thyroid cancer and pancreatic cancer to establish that the genetically predicted elevation in schizophrenia per standard deviation is linked with the development of thyroid cancer (odds ratio (OR) = 1.5482; confidence interval (CI) = 1.1112–2.1569; *p* = 0.0098), yet not with pancreatic cancer (OR = 0.9709; CI = 0.8010–1.1767; *p* = 0.7632). However, the GWAS sample size of thyroid cancer (2) and pancreatic cancer (3) used in the study seems to be smaller and older. Sakaue et al. (4) have recently revised the genome-wide association study (GWAS) data pertaining to thyroid cancer and pancreatic cancer. This update prompts a reevaluation of the validity of the conclusions drawn in the article by Zhou et al. (1) regarding these types of cancer, as well as considering the possibility that alternative findings may arise upon replacement of the revised data.

Second, the GWAS data meta-analysis by Trubetskoy et al. (5) summarized data from multiple cohorts of different origins, including European, East Asian, African American, and Latino ancestry. However, the population included in the Cancer-related outcomes section of Zhou et al. (1) consists solely of individuals of European ancestry. The presence of diverse ethnicities in the exposure and outcome populations may introduce population stratification bias, potentially impacting the robustness of the study’s conclusions.

Finally, the direction of causality of each instrumental variable on exposure and outcome is crucial to the stability of MR conclusions. If variants that exhibit a stronger association with the outcome than the exposure cannot be excluded from the MR analysis, the accuracy of the results may be compromised by the presence of a reverse causation between the exposure and the outcome. The MR Steiger filtering method may be a better way to reduce this possible bias.

In summary, Zhou et al. utilized MR techniques to suggest a potential causal association between schizophrenia and the incidence of various cancers. These findings are worthy of praise. However, there is room for further improvement in the research methods to make the study more robust.

## Author contributions

ZW: Writing – original draft. DL: Writing – original draft. CZ: Writing – review & editing.
